# Management of paradoxical embolism in a patient with coexisting patent foramen ovale and masked pulmonary arteriovenous fistula: A case report

**DOI:** 10.1097/MD.0000000000019507

**Published:** 2020-04-10

**Authors:** Xiao-hua Liu, Jian-min Yang

**Affiliations:** Department of Cardiology, Affiliated Hangzhou First People's Hospital, Zhejiang University School of Medicine, #261 Huansha Road, Shangcheng District, Hangzhou, Zhejiang Province, China.

**Keywords:** intervention treatment, medical therapy, paradoxical embolism, pulmonary arteriovenous fistula

## Abstract

**Rationale:**

Paradoxical embolism (PE) is an important cause of cryptogenic stroke, particularly in young patients, which usually have a relation with an unexpected route in circulation. Here we report a rare case of cryptogenic stroke carried 2 uncommon malformations.

**Patient concerns:**

A 48-year-old female experienced double neurological events in just 2 months.

**Diagnosis:**

Patent foramen ovale was diagnosed with transesophageal echocardiography and successfully occluded in the first admission due to stroke. In the second admission, chest tomographic angiography found a chordae shadow in the right middle lobe, was the first clue for pulmonary arteriovenous fistula (PAVF), thereafter further confirmed by the enhanced pulmonary computed tomographic angiography.

**Interventions:**

This patient then received intervention occlusion therapy with coils for PAVF under the help of microcatheter. Given the possible native origin of the thrombus in PAVF due to the spiral morphology, dual antiplatelet therapy was prescribed for this patient for the first 3 months to prevent device-related embolism after discharge, and the following single antiplatelet therapy was mandated.

**Outcomes:**

No recanalization was detected on the follow-up enhanced pulmonary computed tomographic angiography (PCTA), no neurological defect event recurred in the 16 months of follow-up.

**Lessons:**

Computed tomograph (CT) deserved more value in screening and depicting the morphology of the PAVF, particular in young adults with no apparent arteriosclerotic risk factor. Microcatheter would be helpful for intervention treatment. Antiplatelet therapy might be adequate in specific patients, yet definitely need more evidence to verify.

## 1 Introduction

Paradoxical embolism (PE) could be responsible for most cryptogenic stroke among young patients which was associated with some patterns of abnormal route between systemic and lung circulation. One of the most common reasons is the patent foramen ovale (PFO), which could explain approximately 5-fold of patients <60 years of age with cryptogenic stroke.^[1]^ Not uncommonly, another malformed connection in pulmonary vascular named pulmonary arteriovenous fistula (PAVF) could be also served as a cause of PE. It was reported that this malformation could render about 30% to 40%, 10% to 38%, 10% of carriers to suffer stroke attack, brain abscess, and pulmonary hemorrhage, respectively.^[2]^ Previous cases on this topic often reported the patients with single PFO or PAVF, and most of them mainly discussed the part of invention treatment, failed to pay more attention to the post-intervention medication use.^[3]^ Herein, we presented a patient with coexisting PFO and PAVF who experienced double cryptogenic stroke incidences. Unlike previous reports, our case would discuss more about the diagnostic workup, intervention and medical treatment strategy, and pathogenetic origin speculation.

## 2 Case presentation

**Figure 1:**
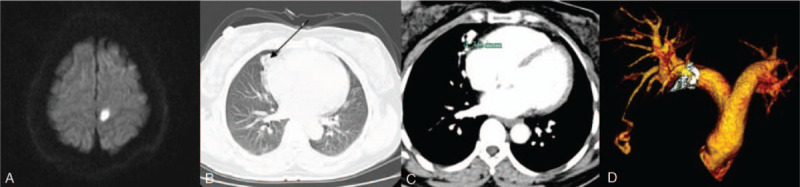
Magnetic resonance imaging detects an acute stroke in left temporal parietal lobe and right parietal lobe (A). Unenhanced chest computed tomograph scan cross-section depicts a chordae shadow in right middle lobe (B, black arrow). Enhanced pulmonary computed tomographic angiography re-confirms a PAVF with a 4.3-mm feeding artery in diameter located in the consistent position as showed on the unenhanced computed tomograph scan (C). Reconstructed image demonstrates its tortuous, spiral tridimensional morphology (D).

This report was approved by the ethics committee of the Affiliated Hangzhou First People's Hospital, Zhejiang University School of Medicine, and the informed consent for publication was obtained from the patient. A 48-year-old female was presented with right hemiparesis and dysphasia, not accompanied by dizziness, nausea, and vomit. The notable medical history was that an acute stroke occurred 2 months ago in the temporal and parietal lobes but without comorbidities of hypertension or diabetes mellitus. Since there was no remarkable atherosclerosis evidence and a large PFO with obvious right-to-left shunting was identified on the transcranial doppler (TCD), this stroke was finally assigned to cryptogenic cause owing to the PFO, thus interventional occlusion was successfully completed. Single antiplatelet treatment with aspirin was prescribed after discharge. Unfortunately, 2 months later, she was admitted to our hospital again with 3 grades level muscle strength on the right lower limb. Brain computed tomograph (CT) scan excluded intracranial hemorrhage, magnetic resonance imaging identified the abnormal signal foci in the left temporal parietal lobe (Fig. 1A), logically diagnosed with stoke.

Subsequently, craniocerebral magnetic resonance angiography and enhanced cervical artery CT scan did not find ischemic evidence, 24 h holter did not detect any episodes of potential stroke-related arrhythmia such as atrial fibrillation as well. Given the negative results of the above inspection, we considered the possibility of PE. Transesophageal echocardiography (TEE) re-confirmed no residual shunt via atrial septal, and all the accomplished workup were reviewed, the chordae shadow in right middle lobe on chest CT scan was noted (Fig. 1B), enhanced pulmonary computed tomographic angiography (PCTA) was therefore performed and confirmed a vascular malformation called PAVF located in the right lung (Fig. 1C and D).

**Figure 2:**
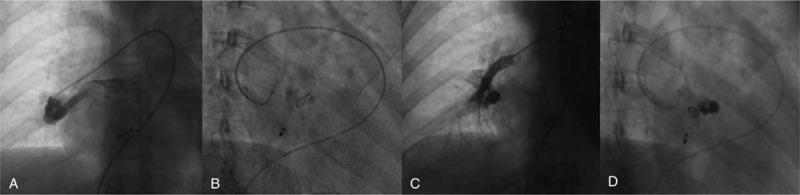
Selected right pulmonary artery angiography shows a PAVF originating from right middle segment pulmonary artery, flowing into right inferior pulmonary vein (A). Microcatheter is delivered into the feeding artery to help plug the distal end of this feeding artery (B). Selected right pulmonary artery angiography verifies the completed occlusion for the PAVF with coils in different views (C and D).

**Figure 3:**
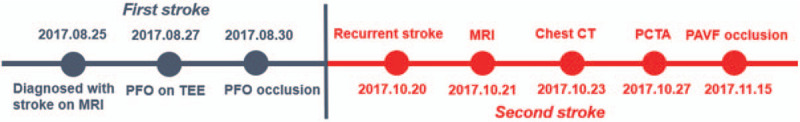
The timeline of diagnosis and therapy for this patient.

The following selected right pulmonary artery angiography verified a PAVF which connected the right middle segment pulmonary artery to the right inferior pulmonary vein (Fig. 2A). The PAVF was successfully plugged with 2 detachable coils (Interlock, Boston Scientific) and 5 undetachable coils (VortX-18, Boston Scientific/G08259, COOK) under the assist of microcatheter (Fig. 2B–D). Dual antiplatelet therapy of aspirin and clopidogrel was prescribed for the initial 3 months after discharge, then switched to single aspirin therapy. No embolic event recurred during the 16 months of follow-up. The process of the diagnosis and therapy is presented in Fig. 3.

## 3 Discussion

PE, as the common cause of undetermined stroke according to the TOAST system,^[4]^ could also develop into devastating complications including stroke, brain abscess, hemothorax despite its occurrence is rare. PFO is a usual defect for asymptomatic PE which could even account for up to 55% of cryptogenic stroke in young adults.^[5]^ The first neurologic event in this patient finally ascribe to the PFO which, to some extent, masked the real pathogenesis of her second stroke which was eventually found a relation to another unexpected entity of PAVF. Indeed, the PAVF was often neglected in clinic practice due to its very low prevalence (2–3 per 100,000 population),^[6]^ partly explained the long hospitalization of the second stroke.

Right-to-left shunts (RLS) on echocardiograph are the representative manifestation for the connected vascular malformation between the right and left circulatory system. TCD and TEE are now accepted as the first recommendation for detecting the RLS with up to 100% sensitivity if combining with Valsalva maneuver.^7^,^8^,^9^ Longer bubble time window (about 15 seconds after contrasts injection) on TCD may help distinguish PAVF-related shunts from the interatrial shunts(about 11 seconds),^10^,^11^ however, these approaches may be more suitable for PFO rather than PAVF because of the ultrasound could be disrupted by the gas in the lung.

Despite PAVF is often a diagnosis of exclusion, a well-designed screening program would be beneficial for shortening the diagnostic time. In this patient, the manifestation of the chest CT scan gave us the first clue of PAVF, whereas TEE failed to detect any hint from heart or pulmonary. Therefore, the unenhanced chest CT scan might be of implication in the preliminary screening for PAVF. The chest manifestation on CT, especially the high-density shadow, should be read with more meticulosity, particularly in young adults with no arteriosclerosis risk factors. Current consensus^[12]^ approved the CT scan as the first-line screening tool rather than echocardiograph, unless the operator has a depth of expertise in echocardiograph. A large-scale study found a higher rate (38 per 100,000 individuals) of PAVF under the low-dose CT screening,^6^,^13^ thus, CT would have a high sensitivity for detecting PAVF. For this patient, the CT provided us the first clue, and the following enhanced PCTA help confirm the diagnosis of PAVF as well as depict the diameter and the tortuous, spiral tridimensional morphology of the PAVF. Consequently, the value of enhanced PCTA on diagnosis and treatment for PAVF deserved more attention, but the increased X-rays exposure should also be treated cautiously.

A diameter of 3 mm of PAVF is reserved as the bound for intervention treatment, this PAVF was measured with a 4.3-mm diameter on the cross-section of enhanced PCTA, and then successfully occluded by coils. Initially, the coils led an indirect occlusion which required the further aggregation of the platelet to complete full obstruction in the feed artery.^[14]^ As a result, about 8% of patients receiving coil occlusion could present with reperfusion in long term follow-up.^[15]^ Insufficient distal occlusion of the feeding artery was found a relation with this reperfusion.^[16]^ In this case, microcatheter was employed in our strategy to help release coils to the distal end of the feeding artery and avoid the venous sac, a total of 7 coils were used to make reliable occlusion of the feeding artery for this PAVF. The following enhanced PCTA confirmed the solid occlusion with no detection of reperfusion 9 months later. Long-term follow-up of PCTA was still necessary for monitoring the possible delayed reperfusion or recanalization.

Reasonably, antithrombotic drugs are essential to prevent recurrent stroke after occlusion. However, a logistic regression by Martin announced that post-occluded antithrombotic therapy was significantly associated with primary (OR 15.21; 95% CI 1.54–150.13; *P* = .02) and recurrent recanalization (OR 6.40; 95% CI 1.31–31.26; *P* = .02).^[17]^ Despite the apprehension for future recanalization, this patient was still mandatory to dual antiplatelet therapy for the first 3 months after discharge and the following single antiplatelet therapy to date. This prescription was based on our speculation for the thrombus’ origin. As there was no definite venous or cardiogenic thrombus, we assumed the pathogenetic thrombus might have a native origin of the PAVF which could be partly explained by its spiral morphology. The location of native thrombus formation was finally filled with sufficient coils, therefore, this patient was prescribed with antiplatelet therapy for preventing the device-related embolism rather than anticoagulation therapy. To some extent, no recurrent neurologic event in follow-up, in turn, confirmed the usefulness of the antiplatelet strategy. Still, we admitted that this strategy was somewhat empirical, clinical trials were needed for further exploring the optimal antithrombotic strategy for these patients.

In conclusion, PAVF, as an uncommon cause of cryptogenic stroke, should be considered for young patients with no obvious risk factors of atherosclerosis. Chest CT or PCTA could be helpful for making the diagnosis and designing treatment strategy. Microcatheter use in intervention treatment could improve success rate. Antithrombotic drugs might be necessary for preventing recurrent stroke, and antiplatelet strategy would be proper in post-interventional medical treatment for certain cases.

## Author contributions

**Data curation:** Xiao-Hua Liu.

**Methodology:** Jian-Min Yang.

**Writing – original draft:** Xiao-Hua Liu.

**Writing – review & editing:** Jian-Min Yang.
